# Seasonal Variation of Aromatic Plants under Cultivation Conditions

**DOI:** 10.3390/plants11162083

**Published:** 2022-08-09

**Authors:** Michalis K. Stefanakis, Charikleia Papaioannou, Vaia Lianopoulou, Eleni Philotheou-Panou, Anastasia E. Giannakoula, Diamanto M. Lazari

**Affiliations:** 1 Laboratory of Plant Physiology, Department of Agriculture, International Hellenic University, 54700 Sindos, Greece; 2 Department of Chemistry, University of Crete, Voutes, 71003 Heraklion, Greece; 3 Laboratory of Genetics, Department of Biology, University of Patras, 26504 Patras, Greece; 4 Laboratory of Pharmacognosy, Faculty of Health Sciences, School of Pharmacy, Aristotle University of Thessaloniki, 54124 Thessaloniki, Greece

**Keywords:** Lamiaceae, volatile compounds, chemical composition, GC-MS, crop yield

## Abstract

In this study, five plant species, members of the Lamiaceae family, namely *Salvia officinalis* L., *Salvia rosmarinus* Spenn, *Mentha × piperita* L., *Mentha spicata* L. and *Origanum vulgare* subsp. *hirtum* (Link) Ietswaart, were studied for the influence of harvesting time on the herb crop yield, the volatile compounds (EOs) content/yield and their chemical composition. EOs were isolated by means of hydro-distillation from different plant parts at different growth stages. Their components were analyzed by gas chromatography coupled with mass spectrometry (GC-MS). The highest yields of EOs were obtained at the full flowering stage and important changes were observed in their composition. The fluctuations in the percentage composition of the major compounds in the EOs, throughout harvesting time, were observed at camphor/α-thujone for *S. officinalis*, camphor/1,8-cineole for *S. rosmarinus*, linalool/linalyl acetate and carvone/limonene for *M. × piperita* and *M. spicata*, respectively. The chemotype of *O. vulgare* subsp. *hirtum* was identified as carvacrol. The optimization of harvesting time could lead to increased crop production and better EOs quality control, with numerous industrial benefits upon the commercial production of such products.

## 1. Introduction

Since ancient times, spices, aromatic herbs and plants played an important role in the life of local communities due to their therapeutic and culinary properties. Among the aromatic plants growing in Greece, the Lamiaceae family is one of the most important, with its members scattered in various locations and nearly all types of vegetation, counting many endemic species, and with most of their plant parts producing essential oils (EOs) of great scientific and economic interest. The commercial value of an aromatic and/or medicinal plant could be reflected by the composition of its EOs [1]. Bearing this in mind, there have been several attempts to establish the stage of development and growing period at which plants produce the best quantity and quality of EOs. In nature, EOs play an important role as secondary metabolites in the protection of plants as antibacterial, antiviral, and antifungal agents, as well as insecticides and substances against herbivory [2]. According to the European Pharmacopoeia, an EO is an ‘odorous product, usually of complex composition, obtained from a botanically defined plant raw material by steam distillation, dry distillation, or a suitable mechanical process without heating. EOs are aromatic oily liquids comprising natural complex mixtures such as hydrocarbons and oxygenated compounds [3]. Their composition may vary considerably among plant species and varieties, or even within the same variety, due to different geographical origins. The factors influencing EOs composition within the same species/variety are the soil type and the environmental conditions, such as temperature, precipitation, relative humidity, day length, and light intensity. All these are essential variables when choosing the optimum sowing time, harvest time, methods of drying and component analysis to reach the best qualitative and quantitative composition of EOs [4,5,6,7,8,9,10].

Studies have already shown that EOs and their main components can play an important role in the biological activity from which food chemistry and pharmaceutics can benefit equally. Nowadays several EOs are recognized as safe substances (ESO, GRAS-182.20) by the Food and Drug Administration (2005) [11] as some contain compounds that can be used as antibacterial additives [12]. Lamiaceae plants are now cultivated worldwide as culinary and medicinal herbs. Due to their components, these aromatic plants are used in the perfume and cosmetic industries, as well as for medicinal and pharmaceutical purposes [13]. The EOs exhibit a wide spectrum of antioxidant, antimicrobial, antiseptic, antifungal, antibacterial, cytotoxic/anticancer, antigenotoxic, antimutagenic and antiviral properties and/or are acting as antiparasitic or disinfectants [8,14,15,16,17,18].

In the current study, the presence of monoterpenes and sesquiterpenes characterized the composition of the EOs of the plants studied. The main constituents of *Origanum vulgare* subsp. *hirtum* (Link) Ietswaart (oregano) are three biosynthetically related compounds: γ-terpinene, *p*-cymene and either thymol or carvacrol depending on its chemotype [3,4,19]. The chemical composition of EOs in *Mentha* species *Mentha* *×* *piperita* L. (peppermint) and *Mentha spicata* L. (spearmint) presents four different chemotypes: the first one is characterized by the dominant occurrence of linalool, the second shows high levels of carvone and dihydrocarvone and the third one presents high percentages of piperitone oxide and/or piperitenone oxide, while the fourth chemotype has only EOs which are rich in menthone, isomenthone and pulegone [20,21,22,23]. Two major types of oil have been identified in *Salvia rosmarinus* Spenn. (syn. *Rosmarinus officinalis* L.: rosemary) plants. The first type of oil contains more than 40% of 1,8-cineole, while in the second one, equal ratios of 1,8-cineole, α-pinene, and camphor have been detected. There are also a few other types of rosemary oil cited in the literature that are rich in verbenone and borneol or in myrcene [7,24]. Finally, in *Salvia officinalis* L. (sage) the predominant ingredients are the monoterpenes α- and β-thujone, 1,8-cineole and camphor. In some cases, linalool, β-pinene, limonene or (*Z*)-sabinyl acetate are the prevailing substances.

Considering the above-mentioned aspects, this study aimed to evaluate and compare the chemical composition of EOs of rosemary, sage, peppermint, spearmint and oregano, growing under cultivation conditions, and the quantitative and qualitative composition of different plant parts. The aforementioned subspecies are popular herbal teas and their EOs can be used as mentioned for different purposes. By studying EOs composition at different plant vegetation phases under cultivating conditions, we expand our scientific knowledge on these plants with the purpose of improving the development of commercial cultivation of these herbs and their exploitation as industrial crops in countries with a similar Mediterranean climate.

## 2. Results

### 2.1. Yield of Essential Oils

**Figure 1:**
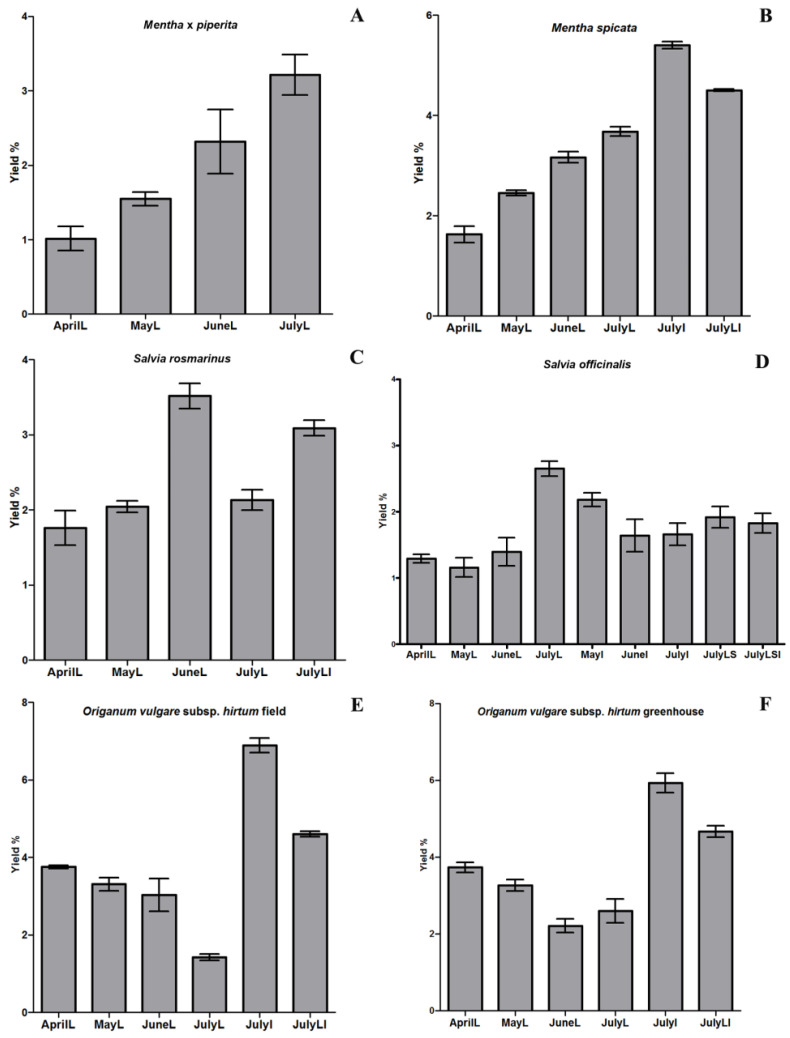
Essential oils’ yield of the studied plant species, collected monthly from April through July, using Kruskal–Wallis multiple comparison tests (L: leaves, I: inflorescences, LI: leaves and inflorescences, LS: leaves and stems, LSI: leaves, stems and inflorescences, added after the name of the collection month). (**A**) *M. × piperita* from fresh leaves (*p* = 0.016), (**B**) *M. spicata* from fresh leaves and/or inflorescences, (*p* = 0.005), (**C**) *S. rosmarinus* from fresh leaves and inflorescences (*p* = 0.013), (**D**) *S. officinalis* from fresh leaves, inflorescences and stems (*p* = 0.003), (**E**) *O. vulgare* subsp. *hirtum* from fresh leaves and/or inflorescences collected from the field (*p* = 0.006) and (**F**) *O. vulgare* subsp. *hirtum* from fresh leaves and/or inflorescences collected from the greenhouse (*p* = 0.006).

The seasonal changes, as well as the type of collected tissue, affected the EO yield, as shown in Figure 1. In addition to that, the EO content distribution between the spring and summer months was uneven.

As shown in Figure 1A, seasonal changes affected the peppermint oil yield and the distribution of the content of EOs was also uneven between the spring and summer months. The lowest amount of oil in *M. × piperita* was found in April (1.01% ± 0.16), and the oil content increased significantly (*p* < 0.05) in July reaching 3.22% ± 0.27.

*M. spicata* showed a significantly higher (*p* < 0.05) EO content in the month of July when the plants were in full bloom and inflorescences were analyzed (5.40% ± 0.07), than that in the month of April (1.63% ± 0.16), when the plants have reached the end of their growth cycle (Figure 1B).

The lowest amount of EO in *S.* *rosmarinus* was found in April (1.76% ± 0.23), while it increased significantly (*p* < 0.05) in June (3.52% ± 0.17) (Figure 1C). The highest yield (3.52%) was obtained for leaves collected at the flowering stage. In *S. officinalis* (Figure 1D), the content of the EO samples analyzed varied significantly across the collection months for leaves, inflorescences and stem tissues (χ^2^ = 23.23, df = 8, *p* = 0.003). More specifically, the yield of leaf oil was lower (1.16% ± 0.14) than the oil yield of inflorescences samples (2.18% ± 0.10) in May and higher in July (JulyL: 2.65% ± 0.11, JulyI: 1.66% ± 0.17).

The content of the EO of both field (Figure 1E) and greenhouse (Figure 1F) *O. vulgare* subsp. *hirtum* samples varied significantly across the collection months for leaves, inflorescences, and leaves/inflorescences tissues analyzed (*p* = 0.006 in both cases). The essential oil yield of the field samples was higher than that of greenhouse samples in April (3.75% ± 0.05 and 3.73% ± 0.14, respectively), May (3.31% ± 0.17 and 3.27% ± 0.15, respectively) and June (3.03% ± 0.42 and 2.21% ± 0.18, respectively) when leaves were analyzed, and in July, when inflorescences (6.89% ± 0.19 and 5.90% ± 0.25, respectively) were analyzed.

### 2.2. Volatile Oil Yield and Composition

The analysis of *M. × piperita* L. oil samples led to the identification of twenty-five compounds, representing 94.2 to 97.1% of the total amount (Table 1).

**Figure 2:**
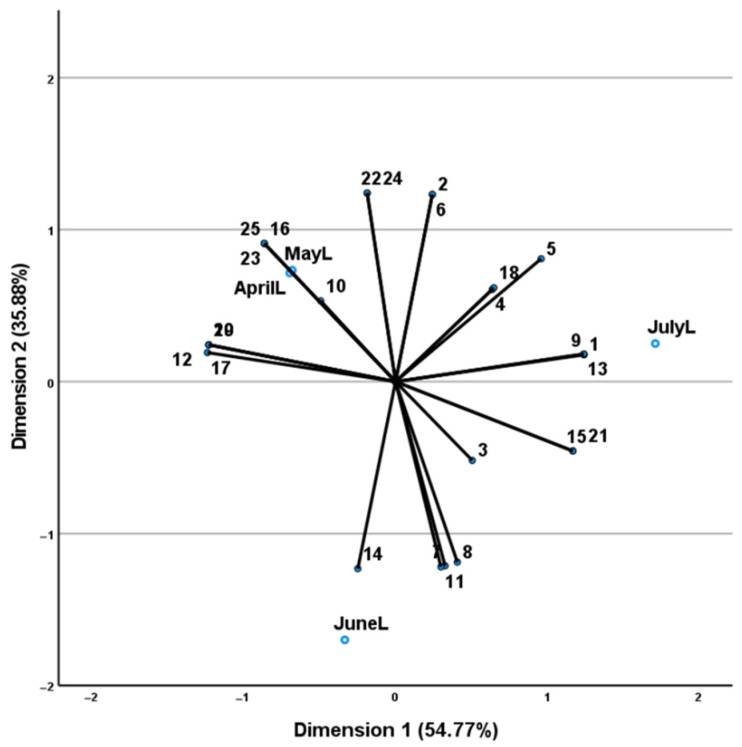
CATPCA analysis of the *M. × piperita* leaf tissue samples (coded as name of collection month followed by L for leaves) based on their essential oil compounds (numbers of which correspond to those of Table 1). Axes 1 and 2 accounted for 90.66% of the total variability and the total value of Cronbach’s alpha (alpha = 0.996) indicated a high level of internal consistency.

The oxygenated monoterpenes were the most abundant (81.9–91.8%), followed by the sesquiterpene hydrocarbons (1.3–10.6%). The main constituent was linalool (39.1–61.1%), followed by linalyl acetate (15.3–32.8%). The results from CATPCA analysis (Figure 2) (constructed using axes 1 and 2) accounted for 90.66% of the total variability and the total value of Cronbach’s alpha (alpha = 0.996) based on the total eigenvalue indicated a high level of internal consistency. Three groups were identified by the dimension 1 (54.77%) and the dimension 2 (35.88%) axes.

The first one included the samples collected in the spring months (April and May), and was characterized by a higher percentage of linalyl acetate, germacrene-D, β-caryophyllene, carvacrol, α-terpineol, octan-3-yl acetate and 1,8-cineole when compared with the summer months and by compounds expressed only in the spring (viridiflorol, α-caryophyllene, terpin-4-ol and linalool oxide); the second cluster included samples collected in June and revealed a higher percentage of linalool, camphor, (*Z*)-ocimene and (*E*)-ocimene, while the third group included samples collected in July, and showed higher percentages of limonene, (*E*)-4-thujanol, geranyl acetate, (*Z*)-pinocamphenone, and the unique compounds β-pinene and (*E*)-pinocarveol.

The analysis of *M. spicata* oil samples allowed the identification of thirty-five components, ranging from seventeen to thirty components depending on the month of collection and the collected tissue, representing 94.6–99.5% of the total oil composition (Table 2).

**Figure 3:**
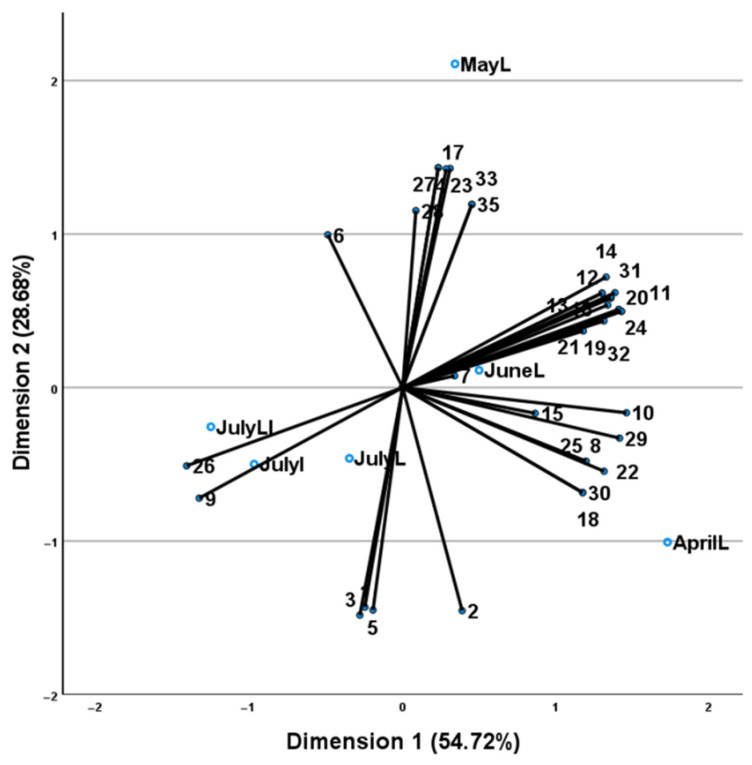
CATPCA analysis of the *M. spicata* leaves and/or inflorescences’ samples (coded as name of collection month followed by L for leaves, I for inflorescences and LI for leaves and inflorescences) based on their essential oil compounds (numbers of which correspond to those of Table 2). Axes 1 and 2, accounted for 83.40% of the total variability and the total value of Cronbach’s alpha (alpha = 0.994) indicated a high level of internal consistency.

The essential oil contained 76.0–83.5% oxygenated monoterpenes, 6.2–19.7% monoterpene hydrocarbons and 1.0–4.6% sesquiterpene hydrocarbons, with the main essential oil constituent being carvone, which increased gradually in the leaves’ tissue from April to July (54.6–65.3%), having the highest concentration in the inflorescence stage during the month of July (73%). Other constituents with high concentrations were limonene (4.7–17.1%), 1,8-cineole (3.2–6.2%), (*Z*)-sabinene hydrate (0.9–7.4%), and dihydrocarveol (0.0–3.4%). CATPCA analysis (Figure 3), constructed using axes 1 and 2, accounted for 83.40% of the total variability and the total value of Cronbach’s alpha (alpha = 0.994) based on the total eigenvalue indicated a high level of internal consistency.

Four groups were identified by the dimension 1 (54.72%) and the dimension 2 (28.68%) axes. The first one included the samples collected in the month of April and was characterized by the higher percentage of 1,8-cineole, dihydrocarvone, germacrene-D, isodihydrocarveol acetate, (*Z*)-carveyl acetate and the unique constituent camphor. The second group included the samples collected in May and was characterized by the higher percentage of (*Z*)-sabinene hydrate, β-bourbonene, (*E*)-carveol, octan-3-ol, oct-1-en-3-ol and the unique constituents octan-3-yl acetate and geraniol. The third group comprised the samples collected in June and was positioned closer to the centroid (0,0), drawn by the constituents present in high concentrations (carvone, limonene, 1,8-cineole, (*Z*)-sabinene hydrate, dihydrocarveol, carvacrol, β-bourbonene, dihydrocarvone and isodihydrocarveol acetate) which were scattered throughout the plot, but was closer to its unique constituent α-terpinene. The final group comprised the samples (leaves and inflorescences) collected in July and was positioned closer to the constituents carvone and limonene, which was expected since these samples present the highest concentration of the two constituents.

Seasonal variations in the composition of essential oils obtained from *S. rosmarinus* samples are shown in Table 3. The analysis of *S. rosmarinus* samples’ oils allowed the identification of 16 compounds, accounting for 89.1–92.8% of the total amount. The monoterpenes dominated, with percentages varying from 61.5–89.1% for the oxygenated and from 0.0–34.9% for the hydrocarbons.

**Figure 4:**
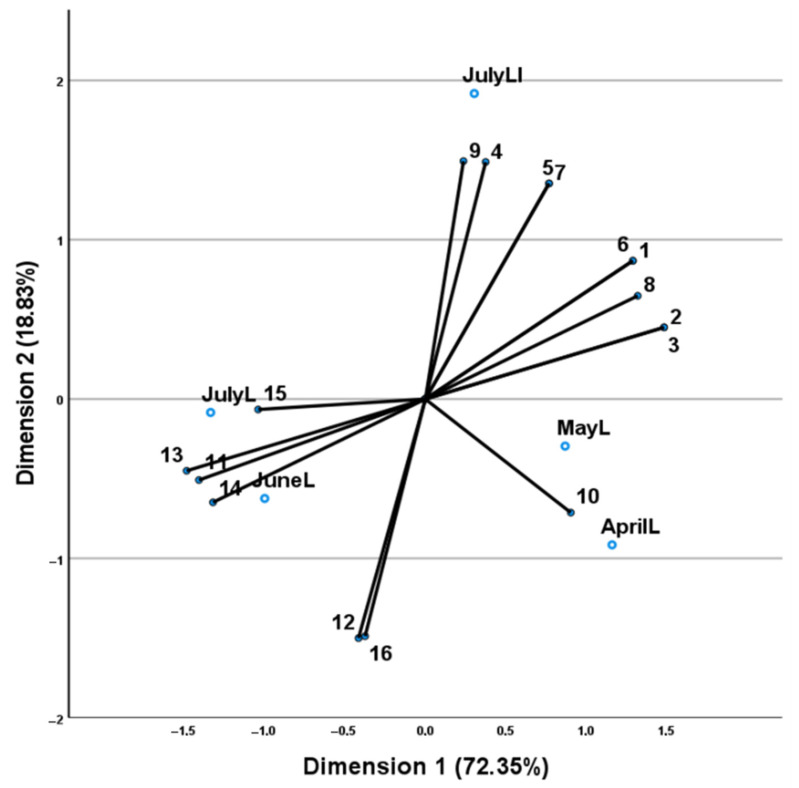
CATPCA analysis based on the chemical composition of *S. rosmarinus* leaf and inflorescences’ essential oils. Distribution of samples (coding the collection months followed by L for leaves’ tissue and LI for leaves and inflorescences) based on the distribution of variables (numbers of which correspond to those of Table 3), indicates a clear separation of the leaves’ samples collected in the spring months (April, May) from those collected during the summer (June, July), in the first-dimension axis.

The most abundant constituent was camphor (29.8–52.6%), followed by 1,8-cineole, borneol and limonene. CATPCA analysis, based on *S. rosmarinus* essential oil data revealed a total Cronbach’s alpha of 0.994 which indicates a high level of internal consistency for our scale. Figure 4 shows the relative position of the sampling months in the discriminant space in relation to a biaxial system.

First dimension axis accounted for 72.35% of the total variance and clearly discriminated the leaves samples collected in the spring months (April and May) from those collected during the summer (June and July). The leaves’ samples collected in April and May were characterized by high percentages of 1,8-cineole and monoterpene hydrocarbons expressed mainly in the spring months (α-pinene, β-pinene, camphene, α-phellandrene, limonene, β-myrcene), while those collected in June and July were characterized by high percentages of oxygenated monoterpenes: camphor, borneol, verbenone, α-terpineol, carvone and isobornyl acetate.

The analysis of *S. officinalis* samples’ oils allowed the identification of 19 components, accounting for 89.3 to 98.2% of the total amount as shown in Table 4, with the oxygenated monoterpenes dominating (56.6–78.6%), followed by the sesquiterpene hydrocarbons (5.2–21.9%) and the oxygenated sesquiterpenes (1.9–10.8%).

The composition analysis of the volatile compounds of *S. officinalis* samples indicated that its EOs belong to the thujone chemotype (α-thujone 19.3–31.6%). The present data also indicated the presence of a high content of camphor (4.9–25.8%), 1,8-cineole (3.3–23.2%) and bornyl acetate (4.0–22.5%).

**Figure 5:**
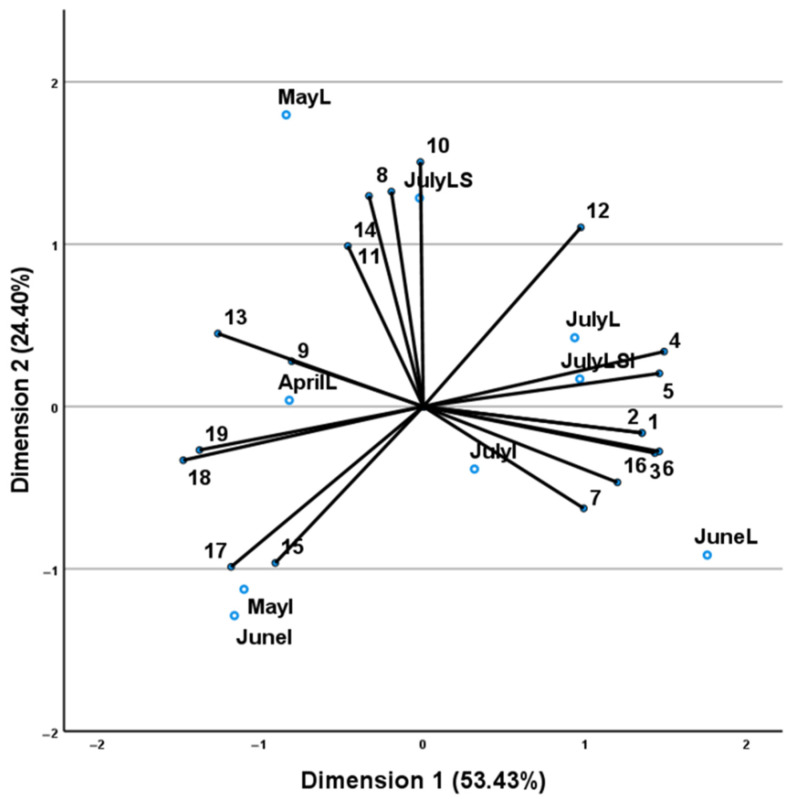
CATPCA analysis of *S. officinalis* samples (L: leaves, I: inflorescences, LS: leaves and stems, LSI: leaves, stems and inflorescences, following the name of the collection month), based on their essential oil compounds (numbers of which correspond to those of Table 4). Axes 1 and 2 accounted for 77.83% of the total variability and the total value of Cronbach’s alpha (alpha = 0.984) indicated a high level of internal consistency.

The data obtained from both leaves, inflorescences and stems across the collection months versus EOs components were evaluated using CATPCA analysis (Figure 5).

The CATPCA (constructed using axes 1 and 2) accounted for 77.83% of the total variability and the total value of Cronbach’s alpha (alpha = 0.984) based on the total eigenvalue, indicating high levels of internal consistency. The first axis (53.43%) divided the leaf samples into two groups. The first (Group I) comprised the samples collected in the spring months (AprilL and MayL) and the second (Group II), the samples collected during the summer months (JuneL and JulyL). The first axis also divided the inflorescence samples into two groups, Group III comprising the inflorescences collected in May and June (MayI and JuneI) and Group IV comprising the ones collected in July (JulyI). Finally, the second axis (24.40%) revealed two groups comprising the combined analyses. The first one (Group V) contained the analysis results of leaf-stem samples and the second (Group VI) the analysis results of leaves-stems-inflorescences samples, collected in July (JulyLS and JulyLSI, respectively). Group I was characterized by high percentages of α-thujone, β-thujone, α-caryophyllene, viridiflorol and borneol. Group II was characterized mainly by high percentages of camphor, 1,8-cineole, β-myrcene, limonene, γ-terpinene and the constituents observed only in the summer months (*E*)-cinnamyl alcohol, β-pinene, camphene and α-pinene. Inflorescences Group III was formed under the influence of the oxygenated monoterpene bornyl acetate, the oxygenated sesquiterpene viridiflorol and the sesquiterpene hydrocarbons α-caryophyllene and β-caryophyllene, while inflorescences group IV position was determined by the positions of its main constituents (1,8-cineole, α-thujone, camphor and bornyl acetate). Finally, the JulyLS samples’ position (GroupV) was highly impacted by its main component which was α-thujone and the JulyLSI samples’ position was the result of the influence of the primary components which were α-thujone, 1,8-cineole, camphor, bornyl acetate, α-caryophyllene, β-caryophyllene and borneol.

The analysis of *O. vulgare* subsp. *hirtum* oil samples revealed 10 compounds, accounting for 93.2–98.7% (for the field-grown samples–OVHF) and 11 compounds, accounting for 94.0–97.4% (for the greenhouse-grown samples–OVHG) of the total amount (Table 5 and Table 6).

The oxygenated monoterpenes were the dominant compounds, varying from 85.3–92.0% for OVHF and from 75.3–91.1% for OVHG, followed by monoterpene hydrocarbons 5.7–11.2% (OVHF) and 0.0–18.7% (OVHG).

The most abundant constituent of *O. vulgare* subsp. *hirtum* oils was carvacrol with a percentage of 82.1–90.9% (OVHF) and 74.5–87.2% (OVHG), followed by γ-terpinene (OVHF: 2.9–5.9%, OVHG: 0.0–10.0%) and *p*-cymene (OVHF: 1.2–6.1%, OVHG: 0.0–12.4%). The content of the rest of the oil components was significantly lower than those of carvacrol, γ-terpinene and *p*-cymene, since the latter compounds were present in a total exceeding 90%.

**Figure 6:**
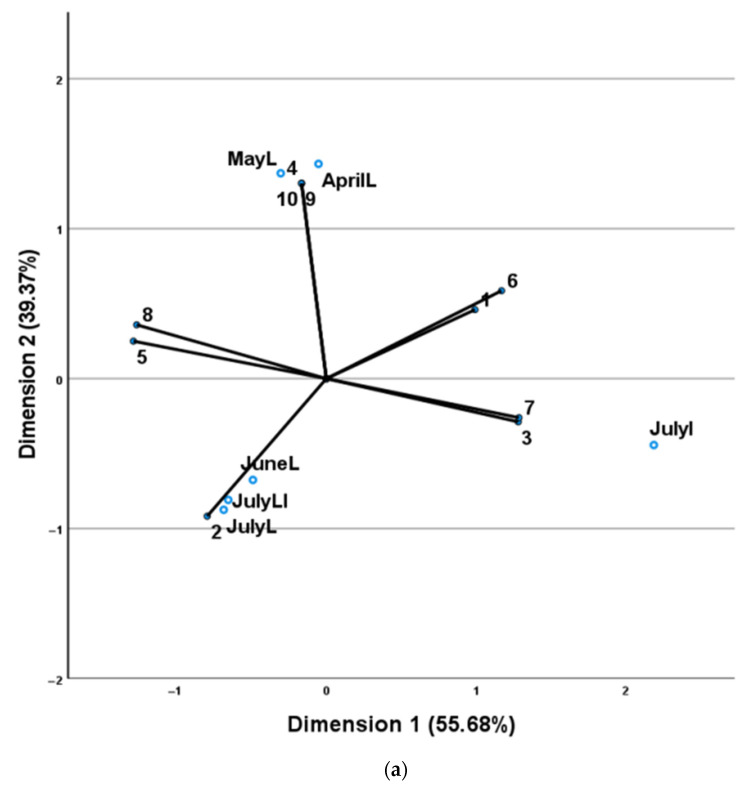

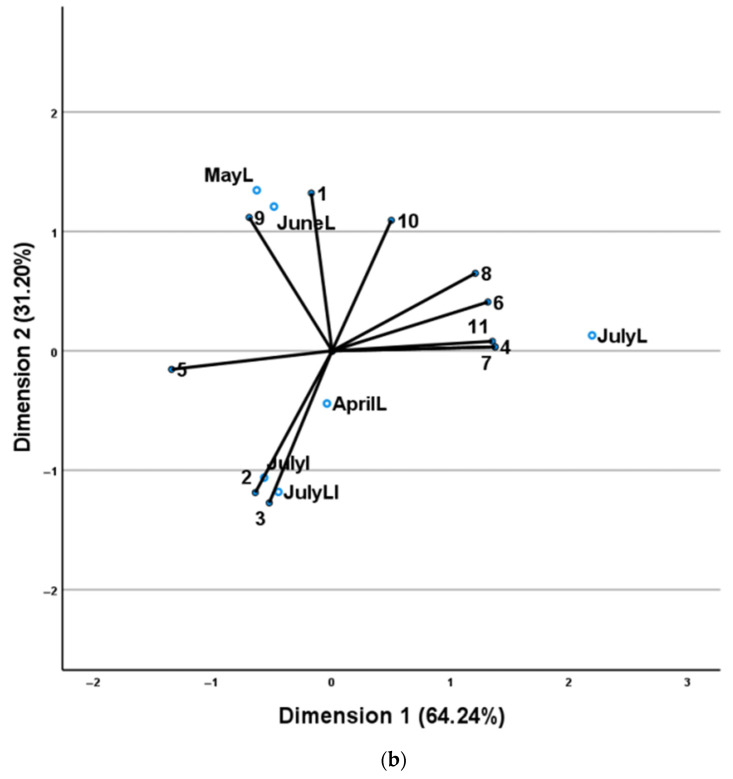
(**a**) CATPCA analyses of chemical composition of *O. vulgare* subsp. *hirtum* leaf (L), inflorescences (I) and leaf-inflorescence (LI) oils, for samples collected (**a**) from the field (OVHF). (**b**) From the greenhouse (OVHG). Distribution of variables (component codes corresponding to those of Table 5 and Table 6) determined the distribution of samples.

The data obtained from the analysis of *O. vulgare* subsp. *hirtum* leaf oil samples from both culture systems, field and greenhouse, were evaluated by CATPCA analyses (Figure 6a,b, respectively).

The OVHF CATPCA analysis constructed using axes 1 and 2 accounted for 95.05% of the total variability and the total value of Cronbach’s alpha (alpha = 0.994), based on the total eigenvalue, indicated a high level of internal consistency. The second axis (39.37%) divided the samples into two groups: the spring leaf samples (Group I) and the summer leaves and inflorescences’ samples (Group II). Group I was characterized by higher percentages of γ-terpinene, α-caryophyllene, germacrene A, thujole, 1,8-cineole and octan-3-one. Group II was characterized by higher percentages of carvacrol, *p*-cymene, β-myrcene and thymol.

The OVHG CATPCA analysis constructed using axes 1 and 2 accounted for 95.43% of the total variability and the total value of Cronbach’s alpha (alpha = 0.995), based on the total eigenvalue, indicated a high level of internal consistency. The first (64.24%) and second axes (31.20%) divided the samples into three groups. The first group (Group I) comprised the May and June leaves’ samples. The second (Group II) comprised the April leaves and the July inflorescences and leaf-inflorescence samples and the third (Group III) the July leaves’ samples. Group I was characterized by high percentages of α-caryophyllene, germacrene A, and octan-3-one. Group II was characterized by high percentages of *p*-cymene, β-myrcene and γ-terpinene and Group III by high percentages of carvacrol, thujole, 1,8-cineole, caryophyllene oxide and thymol.

**Figure 7:**
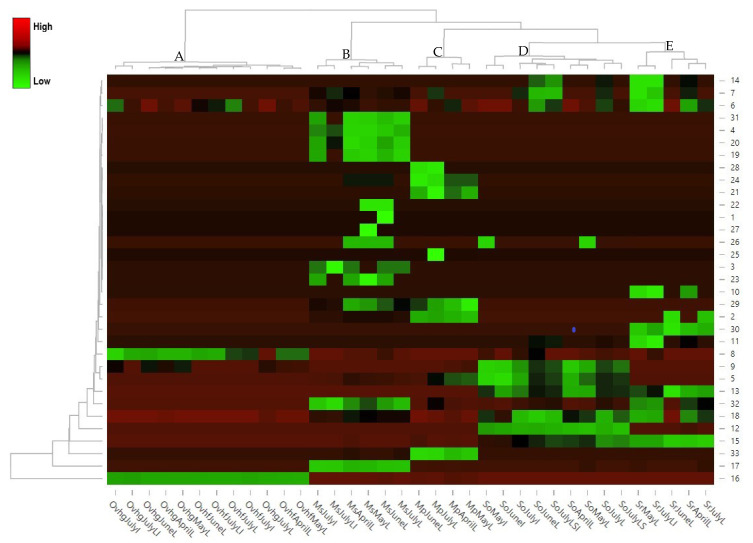
Hierarchical cluster analysis (HCA) and heatmap of all the essential oils’ data combined, based on their volatile compounds. Five clusters were formed. Cluster A comprised the *O. vulgare* subsp. *hirtum* oil samples, both from field and greenhouse, cluster B comprised the *M. spicata* oil samples, cluster C the *M. × piperita* oil samples, and clusters D and E comprised the *S. officinalis* and *S. rosmarinus* oil samples, respectively. Heatmap cells are colored according to the percentages of the main volatile compounds.

Hierarchical cluster analysis (HCA) (Figure 7) based on all the essential oils’ data combined, revealed, the formation of five distinct clusters, each one comprising the different harvesting periods of one distinct species. In the Heatmap, within each group, there was an obvious distinction in the EO composition among the different harvesting months of each plant species, which is presented by the difference in the color scheme. Cluster A comprised the *O. vulgare* subsp. *hirtum* samples, both from field and greenhouse, which presented high similarity in their EO content and composition. Cluster B comprised the *M. spicata* samples. Among them, those harvested in July and in particular JulyI and JulyLI (meaning those where inflorescences were analyzed), which presented the higher oil species’ yield, were the most distinct in the group, forming a separate sub-clade. Cluster C comprised the *M. × piperita* samples, which were separated into two sub-clusters. The first one included those harvested during the summer months, while the second included the samples harvested during spring, emphasizing the quota increase of the EO components as we go from spring to summer. Cluster D comprised the *S. officinalis* samples, with samples MayI and JuneI (when the inflorescences collected in May and June were analyzed) forming a unique sub-cluster (in accordance with CATPCA analysis), mostly due to their quantitative composition of the main constituents. These samples present lower percentages of α-thujone, camphor and 1,8-cineole, while having the highest percentage of bornyl acetate, among the different harvesting periods and plant parts studied. Lastly, cluster E comprised the *S. rosmarinus* samples, with SrMayL and SrJulyLI forming a different sub-clade, due to higher percentages of β-myrcene, β-pinene, α-phellandrene, α-pinene, camphene, 1,8-cineole and limonene, while having lower percentages of borneol, compared to the other samples.

## 3. Discussion

The essential oil amount and composition in aromatic plants are highly dependent on two parameters. One is the soil type; if it is not suitable it may impact the plant growth and essential oil composition (loamy sand is proposed as the most appropriate) [25] and the other is the developmental stage of a plant. Therefore, harvesting time is a crucial factor, that influences the quantity/quality of essential oil. The results of the current study showed that the essential oil yield and content distribution of the studied species were uneven between the spring and summer months.

Analyses of *M. × piperita* essential oil showed that proceeding from spring to summer the yield presented a significant increase. Its essential oil contained mainly linalool and linalyl acetate, with the percentages fluctuating between the spring and summer months. In the literature, it is clearly indicated that the monoterpenes menthone and menthol are considered the major components of the chemical composition of *M. × piperita* [26,27,28]. However, the classification of the genus *Mentha* is very complex and still controversial [29]. The complexity of its taxonomy could be due to different factors namely variation in basic chromosome number, interspecific hybridization, and polymorphism in morphology. The chemotypes’ composition of the *Mentha* species’ essential oil, besides their genetic background, depends on many factors including geographical area, harvest period, climatic conditions and soil characteristics [30]. The mint species is characterized by genetic variability and consequently the presence of many essential oil chemotypes for the same species [31,32]. *Mentha × piperita* L. is a natural interspecific hybrid of spearmint (*M. spicata* L.) and water mint (*M. aquatica* L.). The most common major compounds found in peppermint essential oil are menthol and menthone [33]. However, considering *M. × piperita* essential oil, different studies have shown a high variability derived from the existence of numerous chemotypes, including the carvone chemotype [32,34,35,36,37] and the linalool chemotype [32,38,39,40,41,42,43,44]. Therefore, the linalool chemotype of *M. × piperita* presented in our study has been reported before. Finding new compounds and divergent chemotypes could lead to the evolution and diversity of plants [45] and further shed light on their complex classification. It is assumed that essential oil yield and composition in the medicinal and aromatic plants are generally related to the genetics, climate conditions, altitude and topography of the harvest site [46].

*M. spicata* also presented a gradual increase per month of collection. The least amount of oil was detected in samples collected in April, during the final stages of the plants’ growth cycle, while the peak in oil production was observed in July when the inflorescences were at full bloom stage. Our results agree with those of [23,47], who also reported higher EOs yield of *M. spicata* samples during the summer months. The essential oil comprised mainly oxygenated monoterpenes, monoterpene and sesquiterpene hydrocarbons, with the main constituent, carvone, following the monthly increase pattern and presenting the highest concentration in the inflorescence stage during the month of July. Many researchers [48] reported that the harvesting season has essential effects on the chemical composition of *M. spicata* essential oil, with the essential oil contents increasing as we go from winter and summer. In addition to carvone, our samples also had high concentrations in limonene and 1,8-cineole, findings that comply with the literature [48,49,50] where the above three were reported as the main *M. spicata* essential oil constituents.

The harvesting time of different *S. rosmarinus* samples significantly affected the EO yield, with the highest yield being acquired from leaves collected at the flowering stage. This agrees with the results of [51,52], where the highest EO percentage was observed in the summer months, during the flowering period. In our study, the monoterpenes (oxygenated and hydrocarbons) dominated, as previously mentioned by Pistelli et al. (2018) [53]. The most abundance in camphor, 1,8-cineole, borneol and limonene, is also supported by previous studies [54,55]. However, it is stated in the literature that the essential oil composition of *S. rosmarinus* samples presents variation, which could be attributed to the different varieties [56,57], growth parameters [58] or seasonal changes [59].

In *S. officinalis*, apart from the seasonal changes, the plant part collected also seems to have affected the yield of EO, since there was an obvious inversion in production of leaf versus inflorescences’ oil between May and July sampling, which complies with Arraiza et al. [60] who also observed that the essential oil yield was higher during initial and full flowering and lower after flowering and in the vegetative stand period. In addition to that, the percentages of most components obtained in our study, comply with previous studies with certain variations. This indicates that except for the essential oil yield, the harvesting region, harvesting season and plant part collection appear to also affect its composition [61,62,63].

In *O. vulgare* subsp. *hirtum* samples, both field and greenhouse, the essential oil yield seemed to decrease in leaves, while going from spring to summer, with the highest percentage appearing in the inflorescences, during the full-grown flower period, which agrees with Węglarz et al. [64], who reported that the essential oil yield in Greek oregano reached its maximum during the full blooming stage (third week of July). Analysis of the composition of the essential oil in both cases revealed the abundance of monoterpenes, primarily oxygenated and then hydrocarbons, with carvacrol and γ-terpinene being the principal constituents (>90% in total). The composition of essential oils of oregano is congruent with literature data [3,65] where carvacrol or thymol coupled with *p*-cymene and γ-terpinene were reported as the major constituents. The significantly lower content of the rest of the oil components contributed to the distinct differences in the scent of the plants [3].

HCA and heatmap analyses, which were employed to group the combined data set from all the studied species through their similarities, according to their EO composition, concluded in the formation of five distinct groups, each one comprising the different harvesting periods of one distinct species. In the dendrogram formed, cluster A was the most distant, while clusters B and C, comprising the *Mentha* species studied, were positioned closer, due to the similarity of their essential oils, which contained monoterpenes (oxygenated and hydrocarbons) and sesquiterpene hydrocarbons. Finally, clusters D and E, comprising the *Salvia* species, were also positioned together in the dendrogram, forming a separate group from the other species. *S. rosmarinus* and *S. officinalis* samples, presented many similarities in essential oil constituents, with higher percentages of β-myrcene, β-pinene, borneol, camphor and 1,8-cineole, which placed them closer together. Additionally, HCA analysis of all studied species, further supported the results of CATPCA, indicating that the essential oil composition varies depending on the harvesting period and the plant part collection.

## 4. Materials and Methods

### 4.1. Plant Material

This study was carried out at the research farm of Alexander Technological Educational Institute of Thessaloniki, Greece (latitude 40°39′26.74″ N; 22°48′28.00″ E). Several individuals, of the five herbs, were collected from natural populations located in Stylia Korinthias, Peloponnese, a region in the south of Greece. The peppermint (*M. × piperita*), spearmint (*M. spicata*), sage (*S. officinalis*) and rosemary (*S. rosmarinus*) individuals were planted in the experimental field of the Institute while oregano (*O. vulgare* subsp. *hirtum* (Link) Ietswaart) individuals were cultivated both in a greenhouse in plastic pots and outdoors. In order to evaluate the seasonal variations in the composition of EOs, sampling of leaves and/or inflorescences, and stems and leaves at the inflorescences stage was conducted monthly during the growing period (April to July). Voucher specimens have been deposited in the Herbarium of the Institute. Plants were harvested using a hedge trimmer by cutting the plants approximately 10 cm above the soil surface.

### 4.2. Growing Conditions

The plants were cultivated on the farm at the beginning of October. The planting density was 30 cm within rows and 70 cm between rows. After the first cut, the plant density increased greatly due to the sprouting of buds on the underground rhizomes and kept as perennial crops for the next cropping season. Fertilization was not applied at the experimental site and the method of drip irrigation was used to limit weed’s access to water.

The soil surface layer at the experimental site is up to 2 m and consists of recent alluvial deposits of the Gallikos River. The contours are between 3 ± 8 m. The farmland is dominated by micaceous minerals. The texture of the soil is characterized as sand since the mineral components of soil are dominated by the sand fraction up to 96%, followed by silt and clay. Among the other physical properties of the soil at the experimental site, we mention the bulk density and the total porosity with values ranging from 1.14–1.69 g/cm^3^, and from 36–55% of the total volume, respectively. The soil moisture content is characterized by a wilting point of 1.0–11.6%, a field capacity of 2.7–17.9% and beneficial moisture available of 63–177 mm. The value of pH is between 6.70–10.20. Wherever the underground water level rises to the surface, the soil is saline (EC = 16.32 mmhos/cm). The content of soil-free CaCO_3_ is low reaching only 0.2–2.9%.

### 4.3. Extraction and Isolation

After collection, the samples were dried in the dark at room temperature (25 °C) for 10 days. The dried parts of the plant (approximately 30 g for each extraction) were hydro-distilled for two hours in a Clevenger apparatus connected to a modified EOs container with a refrigerator (each sample was extracted 3 times). After the completion of the distillation, the EOs were diluted with 2 mL of capillary GC grade pentane and dried over anhydrous sodium sulfate and were subsequently analyzed by GC and stored at 4 °C. The oil content was estimated in ml/100 g (dry weight of plant material).

### 4.4. Analysis of Volatile Components

#### 4.4.1. GC–MS Analysis

The composition of the volatile constituents was established by GC-MS analysis. EOs analysis was performed on a Shimadzu GC-2010-GCMS-QP 2010 mass-selective Quadrupole Mass Spectrometer as a detector with the appropriate data system. The GC was equipped with a Grob-type split-splitless injector the fused silica HP-5 MS capillary column (30 m × 0.25 mm i.d., film thickness 0.25 μm) while was directly coupled to the ion source. Helium was used as a carrier gas with a back pressure of 0.8 Atm. Flow rate 1 mL/min. The injector temperature was 230 °C and operated in split mode (split ratio 1:10), while the GC-MS transfer line and the ion source were set at 300 °C and 230 °C, respectively. The oven temperature was programmed to increase from 50 to 290 °C at 4 °C. The scanning range was 30–700 *m/z*. A GC-MS detection electron ionization system was used with an ionization energy of 70 eV.

#### 4.4.2. Identification of Volatile Components

The relative percentage amounts of the separated compound were calculated from the total ion chromatograph by a computerized integrator. The quantification of the components was based on the total number of fragments (total ion count) of the metabolites, as detected by the mass spectrometer. Arithmetic indices for all compounds were determined according to Van Den Dool and Dec. Kratz [66], using n-alkanes as standards (C_8_–C_40_). The identification of the components was based on the comparison of their mass spectra with those of the NIST21 and NIST107 mass spectral libraries [67,68], and of arithmetic indices with literature data [69]. EOs were subjected to co-chromatography with authentic compounds when available (Fluka and Sigma).

### 4.5. Statistical Analysis

In the present study, we analyzed the yield of essential oils from the different species’ plant parts, collected monthly from April through July, using the Kruskal–Wallis H test. The Kruskal–Wallis test is a nonparametric method that does not assume a normal distribution of the residuals, unlike the analogous one-way analysis of variance. We also examined the interrelationship among months of collection and leaves (L), inflorescences (I), or the combinations of leaves–inflorescences (LI), leaves–stems (LS), leaves–stems–inflorescences (LSI) essential oil’s constituents, for each one of the analyzed species, using Categorical Principal Component Analysis (CATPCA). The primary benefit of using CATPCA rather than traditional PCA is that CATPCA does not assume linear relationships among numeric data, nor does it require assuming multivariate normal data. All the analyses mentioned were conducted using the IBM SPSS Statistics software, Version 27 (IBM 2020). Hierarchical cluster analysis (HCA) was used to assign a set of objects into groups so that the most similar objects are positioned in the same cluster. Cluster analysis based on all the essential oils’ data was performed with the Heatmapper online software [70].

## 5. Conclusions

In the present study, five plant species were analyzed regarding the essential oil content and chemical composition of different plant parts, throughout different harvesting periods (spring and summer). The highest content obtained for all the five species, when leaves and/or inflorescences were analyzed, was during the summer months and especially July, except for *S. rosmarinus*, where the highest yield was obtained during June. Fluctuations in chemical composition were more prominent for *M. × piperita*, *M. spicata*, *S. rosmarinus* and *S. officinalis*, than for *O. vulgare* subsp. *hirtum*, with the latest being the most dissimilar in composition from the other four studied species. Oxygenated monoterpene was the most abundant class of volatile compounds in all species. The main constituents identified were linalool for *M. × piperita*, carvone for *M. spicata*, camphor for *S. rosmarinus*, α-thujone for *S. officinalis* and carvacrol for *O. vulgare* subsp. *hirtum*, which presented a significant increase, going from spring to summer months.

In conclusion, there was a clear seasonal harvesting variation, in the yield and composition of essential oils with the summer months and especially July, being the most suitable for harvesting such plants, regarding either the collection of leaves or inflorescences, as they reach the pick of qualitative and quantitative levels of their components.

These findings could be useful for the efficient selection of the best harvesting season, to obtain a higher oil yield, thus constituting a guide for improving the cultivation and processing conditions of these plant species. Additionally, enrichment of these results with further phytochemical studies, such as investigating their non-volatile composition, could contribute to the development of desired cultivars, in harmony with the requirements of food and cosmetic industries.

## Figures and Tables

**Table 1:** Qualitative and quantitative composition of the Essential oils from *M. × piperita*.

No	Compounds ^a^	AI ^b^	AI ^c^	AprilL	MayL	JuneL	JulyL
1	β-Pinene	973	974	n.d. ^d^	n.d.	n.d.	0.9 ± 0.12
2	β-Myrcene	990	990	1.3 ± 0.02	0.2 ± 0.00	0.1 ± 0.00	0.6 ± 0.01
3	Octan-3-ol	995	988	n.d.	0.1 ± 0.01	0.1 ± 0.01	0.1 ± 0.01
4	*p*-Cymene	1022	1024	0.7 ± 0.10	0.1 ± 0.01	0.1 ± 0.01	0.2 ± 0.02
5	Limonene	1026	1029	0.3 ± 0.19	0.1 ± 0.00	n.d.	2.3 ± 0.50
6	1,8-Cineole	1029	1026	1.3 ± 0.23	0.4 ± 0.15	n.d.	0.5 ± 0.30
7	(*Z*)-Ocimene	1038	1037	0.6 ± 0.09	0.4 ± 0.02	1.4 ± 1.25	0.7 ± 0.07
8	(*E*)-Ocimene	1048	1044	0.2 ± 0.00	0.2 ± 0.00	1.0 ± 0.04	0.8 ± 0.02
9	(*E*)-4-Thujanol	1065	1065	0.4 ± 0.15	0.7 ± 0.01	0.7 ± 0.01	2.0 ± 0.98
10	Linalool oxide	1087	1084	n.d.	0.1 ± 0.00	n.d.	n.d.
11	Linalool	1097	1097	39.1 ± 2.02	46.2 ± 2.13	61.1 ± 1.25	59.8 ± 1.18
12	Octan-3-yl acetate	1124	1120	1.9 ± 0.29	1.6 ± 0.33	1.4 ± 0.43	0.8 ± 0.20
13	(*E*)-Pinocarveol	1135	1135	n.d.	n.d.	n.d.	0.5 ± 0.00
14	Camphor	1141	1141	0.4 ± 0.01	n.d.	1.6 ± 0.00	n.d.
15	(*Z*)-Pinocamphenone	1171	1172	0.3 ± 0.02	0.3 ± 0.01	0.8 ± 0.03	1.7 ± 0.50
16	Terpin-4-ol	1174	1174	0.2 ± 0.00	0.2 ± 0.02	n.d.	n.d.
17	α-Terpineol	1188	1186	3.5 ± 1.20	4.2 ± 1.45	3.2 ± 1.00	2.8 ± 0.75
18	Carvone	1242	1239	n.d.	0.4 ± 0.00	n.d.	0.4 ± 0.00
19	Linalyl acetate	1255	1254	32.8 ± 1.20	25.0 ± 1.00	20.5 ± 1.00	15.3 ± 1.35
20	Carvacrol	1299	1298	5.2 ± 0.08	2.9 ± 0.05	2.4 ± 0.02	1.5 ± 0.30
21	Geranyl acetate	1381	1379	n.d.	n.d.	1.3 ± 0.25	1.5 ± 0.43
22	β-Caryophyllene	1418	1417	3.4 ± 0.20	3.7 ± 1.20	0.2 ± 0.00	1.8 ± 0.70
23	α-Caryophyllene	1452	1454	0.2 ± 0.01	0.4 ± 0.01	n.d.	n.d.
24	Germacrene-D	1480	1484	3.3 ± 1.13	6.5 ± 2.10	1.1 ± 0.07	2.1 ± 0.30
25	Viridiflorol	1592	1592	0.3 ± 0.01	0.6 ± 0.02	n.d.	n.d.
	Total identified (%)			95.3	94.2	97.1	96.3
	Monoterepene hydrocarbons			3.1	1.0	2.6	5.5
	Oxygenated monoterpenes			84.9	81.9	91.8	85.3
	Sesquiterpene hydrocarbons			6.9	10.6	1.3	3.9
	Oxygenated sesquiterpenes			0.3	0.6	1.3	1.5
	Others			-	0.1	0.1	0.1
	Essential oil content (mL/100 g dry weight)			1.01	1.55	2.32	3.22

(a) Compounds are listed in the order of elution from an HP-5 MS capillary column; (b) AI: Arithmetic index determined relative to a homologous series of n-alkanes (C_9_–C_25_) on an HP-5 MS capillary column; (c) AI: Arithmetic index from literature data and Adams (2007) (d) n.d.: not detected.

**Table 2:** Qualitative and quantitative composition of the Essential oils from *M. spicata*.

No	Compounds ^a^	AI ^b^	AI ^c^	AprilL	MayL	JuneL	JulyL	JulyI	JulyLI
1	α-Thujene	931	924	0.1 ± 0.00	n.d. ^d^	0.1 ± 0.00	0.1 ± 0.00	0.1 ± 0.00	0.5 ± 0.01
2	Sabinene	971	969	0.5 ± 0.01	0.2 ± 0.01	0.3 ± 0.01	0.3 ± 0.01	0.3 ± 0.00	0.4 ± 0.01
3	β-Pinene	973	974	0.5 ± 0.01	0.2 ± 0.01	0.3 ± 0.01	0.4 ± 0.01	0.4 ± 0.01	0.8 ± 0.02
4	Oct-1-en-3-ol	979	974	0.1 ± 0.00	0.2 ± 0.01	0.1 ± 0.00	0.1 ± 0.00	n.d.	0.1 ± 0.00
5	β-Myrcene	990	990	0.8 ± 0.01	0.5 ± 0.02	0.6 ± 0.02	0.6 ± 0.02	0.6 ± 0.02	0.9 ± 0.03
6	Octan-3-ol	995	988	0.5 ± 0.01	0.6 ± 0.02	0.5 ± 0.02	0.4 ± 0.02	0.5 ± 0.01	0.6 ± 0.02
7	α-Terpinene	1014	1014	n.d.	n.d.	0.1 ± 0.00	n.d.	n.d.	n.d.
8	*p*-Cymene	1023	1024	0.2 ± 0.00	n.d.	0.2 ± 0.00	n.d.	n.d.	n.d.
9	Limonene	1026	1029	6.7 ± 1.25	4.7 ± 0.90	7.1 ± 1.13	10.9 ± 1.00	12.8 ± 1.20	17.1 ± 1.10
10	1,8-Cineole	1028	1026	6.2 ± 1.00	5.3 ± 097	4.8 ± 0.50	4.8 ± 0.82	3.4 ± 0.90	3.2 ± 0.75
11	(*Z*)-Ocimene	1038	1037	0.3 ± 0.01	0.3 ± 0.02	0.3 ± 0.03	0.2 ± 0.00	0.1 ± 0.00	n.d.
12	(*E*)-Ocimene	1048	1044	0.1 ± 0.00	0.1 ± 0.02	0.1 ± 0.00	n.d.	n.d.	n.d.
13	γ-Terpinene	1057	1054	0.3 ± 0.01	0.3 ± 0.02	0.8 ± 0.05	0.2 ± 0.01	n.d.	n.d.
14	(*Z*)-Sabinene hydrate	1065	1065	5.1 ± 0.80	7.4 ± 0.56	4.6 ± 0.20	3.1 ± 0.20	2.1 ± 0.15	0.9 ± 0.20
15	Linalool	1097	1097	1.6 ± 0.50	0.2 ± 0.01	n.d.	1.7 ± 0.20	n.d.	n.d.
16	(*E*)-Sabinene hydrate	1098	1098	0.2 ± 0.02	0.2 ± 0.01	0.2 ± 0.02	n.d.	n.d.	n.d.
17	Octan-3-yl acetate	1124	1120	n.d.	0.1 ± 0.00	n.d.	n.d.	n.d.	n.d.
18	Camphor	1141	1141	1.0 ± 0.20	n.d.	n.d.	n.d.	n.d.	n.d.
19	Terpin-4-ol	1174	1174	1.4 ± 0.20	1.2 ± 0.20	1.0 ± 0.12	n.d.	0.4 ± 0.00	0.4 ± 0.01
20	α-Terpineol	1188	1186	0.5 ± 0.03	0.5 ± 0.03	0.5 ± 0.03	0.4 ± 0.02	0.3 ± 0.01	0.3 ± 0.02
21	Dihydrocarveol	1192	1192	3.0 ± 0.84	3.4 ± 0.55	2.2 ± 0.92	3.4 ± 0.62	1.7 ± 0.98	n.d.
22	Dihydrocarvone	1194	1200	2.3 ± 0.42	2.0 ± 0.38	1.0 ± 0.55	1.9 ± 0.90	1.0 ± 0.36	0.3 ± 0.06
23	(*E*)-Carveol	1216	1215	0.2 ± 0.01	0.7 ± 0.32	0.2 ± 0.05	n.d.	0.2 ± 0.02	n.d.
24	(*Z*)-Carveol	1229	1226	1.3 ± 0.89	1.0 ± 0.60	0.7 ± 0.22	0.5 ± 0.10	0.4 ± 0.02	n.d.
25	Pulegone	1237	1233	0.2 ± 0.01	n.d.	0.2 ± 0.01	n.d.	0.1 ± 0.00	n.d.
26	Carvone	1242	1239	54.6 ± 0.00	57.1 ± 0.05	64.1 ± 0.10	65.3 ± 0.22	73.0 ± 0.00	70.7 ± 0.45
27	Geraniol	1249	1249	n.d.	0.4 ± 0.02	n.d.	n.d.	n.d.	n.d.
28	Carvacrol	1299	1298	n.d.	0.8 ± 0.20	2.8 ± 1.02	n.d.	n.d.	0.1 ± 0.00
29	Isodihydrocarveol acetate	1327	1326	1.5 ± 0.43	1.4 ± 0.92	1.1 ± 0.82	1.4 ± 0.55	0.7 ± 0.12	n.d.
30	(*Z*)-Carveyl acetate	1361	1365	1.4 ± 0.30	0.7 ± 0.30	n.d.	n.d.	0.2 ± 0.05	n.d.
31	β-Bourbonene	1383	1387	1.8 ± 0.90	1.8 ± 0.80	1.5 ± 0.82	1.1 ± 0.90	0.7 ± 0.26	0.5 ± 0.10
32	β-Caryophyllene	1418	1417	0.8 ± 0.10	0.6 ± 0.12	0.5 ± 0.15	0.3 ± 0.12	0.1 ± 0.00	0.2 ± 0.01
33	(*E*)-β-Farnesene	1455	1454	n.d.	0.2 ± 0.00	0.2 ± 0.01	n.d.	n.d.	n.d.
34	Germacrene-D	1480	1484	2.5 ± 0.55	2.0 ± 0.48	1.4 ± 0.36	0.6 ± 0.10	0.4 ± 0.08	0.3 ± 0.06
35	Viridiflorol	1592	1592	n.d.	0.5 ± 0.20	0.6 ± 0.10	n.d.	n.d.	n.d.
	Total identified (%)			95.7	94.6	98.1	97.7	99.5	97.3
	Monoterepene hydrocarbons			9.4	6.2	9.8	12.8	14.3	19.7
	Oxygenated monoterpenes			80.7	82.5	83.4	82.5	83.5	76.0
	Sesquiterpene hydrocarbons			5.0	4.6	3.7	2.0	1.2	1.0
	Oxygenated sesquiterpenes			-	0.5	0.6	-	-	-
	Others			0.6	0.8	0.6	0.4	0.5	0.6
	Essential oil content (mL/100 g dry weight)			1.63	2.45	3.16	3.68	5.40	4.50

(a) Compounds are listed in the order of elution from an HP-5 MS capillary column; (b) AI: Arithmetic index determined relative to a homologous series of n-alkanes (C_9_–C_25_) on an HP-5 MS capillary column; (c) AI: Arithmetic index from literature data and Adams (2007) (d) n.d.: not detected.

**Table 3:** Qualitative and quantitative composition of the Essential oils from *S. rosmarinus*.

No	Compounds ^a^	A.I ^b^	A.I ^c^	AprilL	MayL	JuneL	JulyL	JulyI
1	α-Pinene	930	932	0.6 ± 0.00	8.7 ± 0.15	n.d. ^d^	n.d.	12.0 ± 0.05
2	Camphene	954	946	0.4 ± 0.01	4.5 ± 0.08	n.d.	n.d.	4.5 ± 0.02
3	β-Pinene	973	974	0.7 ± 0.01	4.5 ± 0.05	n.d.	n.d.	3.0 ± 0.05
4	Octan-3-one	987	979	3.8 ± 0.80	2.4 ± 0.60	n.d.	2.9 ± 0.30	4.0 ± 0.08
5	β-Myrcene	990	990	2.5 ± 0.50	4.9 ± 0.50	n.d.	1.4 ± 0.05	5.5 ± 0.20
6	α-Phellandrene	1002	1002	0.6 ± 0.01	1.6 ± 0.05	n.d.	n.d.	2.0 ± 0.08
7	Limonene	1026	1029	4.2 ± 0.95	6.6 ± 0.55	n.d.	2.5 ± 0.40	7.0 ± 0.40
8	1,8-Cineole	1029	1026	12.0 ± 1.00	15.8 ± 1.10	n.d.	7.9 ± 0.15	14.6 ± 0.90
9	γ-Terpinene	1059	1054	n.d.	n.d.	n.d.	n.d.	0.9 ± 0.01
10	α-Thujone	1102	1101	1.0 ± 0.05	n.d.	n.d.	n.d.	n.d.
11	Camphor	1141	1141	47.5 ± 1.22	29.8 ± 0.98	50.4 ± 1.00	52.6 ± 0.85	30.0 ± 0.28
12	Borneol	1169	1165	8.6 ± 0.75	4.4 ± 0.50	17.1 ± 1.02	7.1 ± 0.20	2.7 ± 0.02
13	α-Terpineol	1188	1186	n.d.	n.d.	6.6 ± 0.36	4.3 ± 0.15	n.d.
14	Verbenone	1205	1204	3.7 ± 0.24	1.7 ± 0.04	6.2 ± 0.50	5.8 ± 0.40	3.0 ± 0.01
15	Carvone	1242	1239	n.d.	1.6 ± 0.05	n.d.	3.1 ± 0.01	n.d.
16	Isobornyl acetate	1288	1287	5.1 ± 0.60	5.8 ± 0.30	8.8 ± 0.08	4.1 ± 0.08	3.6 ± 0.25
	Total identified (%)			90.6	92.3	89.1	91.7	92.8
	Monoterpene hydrocarbons			9.0	30.8	-	3.9	34.9
	Oxygenated monoterpenes			81.6	61.5	89.1	87.8	57.9
	Essential oil content (mL/100 g dry weight)			1.76	2.04	3.52	2.13	3.09

(a) Compounds are listed in the order of elution from an HP-5 MS capillary column; (b) AI: Arithmetic index determined relative to a homologous series of n-alkanes (C_9_–C_25_) on an HP-5 MS capillary column; (c) AI: Arithmetic index from literature data and Adams (2007) (d) n.d.: not detected.

**Table 4:** Qualitative and quantitative composition of the Essential oils parts from *S. officinalis*.

No	Compounds ^a^	A.I. ^b^	A.I. ^c^	AprilL	MayL	JuneL	JulyL	MayI	JuneI	JulyI	JulyLS	JulyLSI
1	α-Pinene	930	932	n.d. ^d^	n.d.	0.8 ± 0.20	0.2 ± 0.01	n.d.	n.d.	n.d.	n.d.	1.0 ± 0.00
2	Camphene	954	946	n.d.	n.d.	1.1 ± 0.02	0.5 ± 0.02	n.d.	n.d.	n.d.	n.d.	1.5 ± 0.04
3	β-Pinene	973	974	n.d.	n.d.	2.5 ± 0.92	0.8 ± 0.01	n.d.	n.d.	0.8 ± 0.02	n.d.	2.6 ± 0.80
4	β-Myrcene	990	990	n.d.	0.3 ± 0.01	2.4 ± 0.01	1.6 ± 0.36	n.d.	n.d.	0.5 ± 0.01	0.6 ± 0.01	1.5 ± 0.02
5	Limonene	1026	1029	n.d.	0.2 ± 0.00	1.2 ± 0.05	1.4 ± 0.10	0.2 ± 0.02	n.d.	0.7 ± 0.00	0.7 ± 0.00	1.5 ± 0.04
6	1,8-Cineole	1029	1026	6.0 ± 1.10	8.0 ± 0.90	23.2 ± 0.90	16.4 ± 0.65	7.7 ± 0.20	3.3 ± 1.13	18.5 ± 0.74	9.9 ± 0.84	19.2 ± 0.90
7	γ-Terpinene	1059	1054	n.d.	0.3 ± 0.01	1.9 ± 0.25	0.6 ± 0.00	0.7 ± 0.00	n.d.	1.0 ± 0.05	0.4 ± 0.01	n.d.
8	(*Z*)-Sabinene hydrate	1065	1065	n.d.	0.5 ± 0.00	0.2 ± 0.00	0.3 ± 0.01	n.d.	n.d.	n.d.	0.3 ± 0.00	n.d.
9	(*E*)-Sabinene hydrate	1098	1098	n.d.	0.3 ± 0.01	n.d.	n.d.	0.3 ± 0.01	n.d.	n.d.	n.d.	n.d.
10	α-Thujone	1102	1101	28.4 ± 1.23	31.6 ± 0.92	23.0 ± 1.56	24.8 ± 1.10	19.3 ± 1.24	21.6 ± 2.02	24.5 ± 0.99	31.3 ± 2.13	25.7 ± 1.00
11	β-Thujone	1112	1112	4.7 ± 1.00	4.9 ± 0.85	4.1 ± 0.45	4.5 ± 0.30	4.6 ± 0.20	3.8 ± 0.15	4.4 ± 0.08	5.9 ± 0.14	4.6 ± 0.10
12	Camphor	1141	1141	16.5 ± 1.20	14.3 ± 0.36	13.2 ± 0.62	25.8 ± 0.84	4.9 ± 1.23	5.5 ± 0.08	9.6 ± 0.33	24.5 ± 1.00	17.6 ± 0.62
13	Borneol	1169	1165	8.1 ± 0.05	7.8 ± 0.42	2.8 ± 0.20	3.3 ± 0.05	3.6 ± 0.00	7.0 ± 0.05	5.7 ± 0.45	3.5 ± 0.30	3.3 ± 0.02
14	Carvone	1242	1239	n.d.	2.5 ± 0.50	n.d.	n.d.	n.d.	n.d.	n.d.	n.d.	n.d.
15	Bornyl acetate	1288	1287	4.0 ± 0.27	4.7 ± 0.50	5.0 ± 0.00	9.2 ± 0.05	22.5 ± 0.02	15.5 ± 0.50	9.1 ± 0.30	10.8 ± 0.55	8.2 ± 0.30
16	(*E*)-Cinnamyl alcohol	1304	1303	n.d.	n.d.	7.4 ± 0.05	1.1 ± 0.02	n.d.	n.d.	5.2 ± 0.02	n.d.	n.d.
17	β-Caryophyllene	1418	1417	6.4 ± 0.20	3.9 ± 0.05	2.3 ± 0.04	2.4 ± 0.01	10.7 ± 0.10	11.7 ± 0.10	5.7 ± 0.02	2.8 ± 0.05	3.0 ± 0.05
18	α-Caryophyllene	1452	1454	9.8 ± 0.97	6.4 ± 0.55	2.9 ± 1.10	3.4 ± 0.20	10.9 ± 0.09	10.3 ± 0.08	5.2 ± 0.00	4.5 ± 0.05	3.6 ± 1.00
19	Viridiflorol	1592	1592	7.3 ± 0.50	9.1 ± 0.30	3.5 ± 0.40	1.9 ± 0.10	8.7 ± 0.10	10.8 ± 0.10	5.5 ± 0.08	2.1 ± 0.10	2.5 ± 0.40
	Total identified (%)			91.1	94.8	97.5	98.2	94.0	89.3	96.4	97.2	95.8
	Monoterpene hydrocarbons			-	0.8	9.9	5.0	0.9	-	3.0	1.7	8.1
	Oxygenated monoterpenes			67.6	74.6	78.9	85.5	62.8	56.6	77.0	86.1	78.6
	Sesquiterpene hydrocarbons			16.1	10.3	5.2	5.8	21.6	21.9	10.9	7.3	6.6
	Oxygenated sesqiterpenes			7.4	9.1	3.5	1.9	8.7	10.8	5.5	2.1	2.5
	Essential oil content (mL/100 g dry weight)			1.29	1.16	1.39	2.65	2.18	1.64	1.66	1.92	1.83

(a) Compounds are listed in the order of elution from an HP-5 MS capillary column; (b) AI: Arithmetic index determined relative to a homologous series of n-alkanes (C_9_–C_25_) on an HP-5 MS capillary column; (c) AI: Arithmetic index from literature data and Adams (2007) (d) n.d.: not detected.

**Table 5:** Qualitative and quantitative composition of the Essential oils from *O. vulgare* subsp. *hirtum* collected from the field.

No	Compounds ^a^	AI ^b^	AI ^c^	AprilL	MayL	JuneL	JulyL	JulyI	JulyLI
1	Octan-3-one	987	979	0.6 ± 0.00	n.d. ^d^	n.d.	n.d.	0.6 ± 0.00	n.d.
2	β-Myrcene	990	990	0.5 ± 0.02	0.3 ± 0.01	0.9 ± 0.20	2.2 ± 0.90	0.4 ± 0.02	1.2 ± 0.50
3	*p*-Cymene	1022	1024	1.2 ± 0.05	1.6 ± 0.05	4.9 ± 1.20	2.5 ± 1.40	6.1 ± 1.00	3.3 ± 1.20
4	1,8-Cineole	1029	1026	0.3 ± 0.00	0.3 ± 0.00	n.d.	n.d.	n.d.	n.d.
5	γ-Terpinene	1059	1054	4.0 ± 0.50	4.0 ± 0.45	5.4 ± 1.00	3.2 ± 0.90	2.9 ± 0.85	5.9 ± 0.55
6	Thujol	1162	1168	0.7 ± 0.03	0.7 ± 0.03	n.d.	n.d.	0.8 ± 0.04	n.d.
7	Thymol	1290	1289	0.1 ± 0.00	0.1 ± 0.00	n.d.	n.d.	2.6 ± 0.33	0.6 ± 0.02
8	Carvacrol	1299	1298	89.3 ± 1.60	90.9 ± 0.80	84.0 ± 1.00	85.3 ± 1.23	82.1 ± 0.90	86.1 ± 0.82
9	α-Caryophyllene	1452	1454	0.6 ± 0.02	0.5 ± 0.02	n.d.	n.d.	n.d.	n.d.
10	Germacrene A	1509	1508	0.2 ± 0.01	0.3 ± 0.01	n.d.	n.d.	n.d.	n.d.
	Total identified (%)			97.5	98.7	95.2	93.2	95.5	97.1
	Monoterpene hydrocarbons			5.7	5.9	11.2	7.9	9.4	10.4
	Oxygenated monoterpenes			91.0	92.0	84.0	85.3	86.1	86.7
	Sesquiterpene hydrocarbons			0.8	0.8	-	-	-	-
	Essential oil content (mL/100 g dry weight)			3.75	3.31	3.03	1.42	6.89	4.60

(a) Compounds are listed in the order of elution from an HP-5 MS capillary column; (b) AI: Arithmetic index determined relative to a homologous series of n-alkanes (C_9_–C_25_) on an HP-5 MS capillary column; (c) AI: Arithmetic index from literature data and Adams (2007) (d) n.d.: not detected.

**Table 6:** Qualitative and quantitative composition of the Essential oils from *O. vulgare* subsp. *hirtum* collected from the greenhouse.

No	Compounds ^a^	AI ^b^	AI ^c^	AprilL	MayL	JuneL	JulyL	JulyI	JulyLI
1	Octan-3-one	987	979	0.7 ± 0.14	1.2 ± 0.60	1.1 ± 0.02	1.0 ± 0.00	n.d. ^d^	0.8 ± 0.02
2	β-Myrcene	990	990	0.4 ± 0.02	0.2 ± 0.01	n.d.	n.d.	2.0 ± 0.83	0.5 ± 0.01
3	*p*-Cymene	1022	1024	2.6 ± 0.83	n.d.	n.d.	n.d.	5.6 ± 1.20	12.4 ± 1.82
4	1,8-Cineole	1029	1026	0.3 ± 0.02	n.d.	n.d.	1.1 ± 0.02	n.d.	n.d.
5	γ-Terpinene	1059	1054	6.6 ± 1.00	6.6 ± 0.95	5.5 ± 0.33	n.d.	10.0 ± 1.60	5.8 ± 0.83
6	Thujol	1162	1168	0.5 ± 0.08	0.7 ± 0.05	0.5 ± 0.01	1.6 ± 0.14	n.d.	n.d.
7	Thymol	1290	1289	0.1 ± 0.00	n.d.	n.d.	0.2 ± 0.00	n.d.	n.d.
8	Carvacrol	1299	1298	83.2 ± 1.50	83.4 ± 1.00	85.0 ± 0.85	87.2 ± 1.00	76.3 ± 1.00	74.5 ± 1.20
9	α-Caryophyllene	1452	1454	1.3 ± 0.75	2.4 ± 0.84	2.2 ± 0.50	1.1 ± 0.22	1.7 ± 0.05	n.d.
10	Germacrene A	1509	1508	1.7 ± 0.60	1.4 ± 0.50	1.4 ± 0.01	1.6 ± 0.04	n.d.	n.d.
11	Caryophyllene oxide	1583	1582	n.d.	n.d.	n.d.	1.1 ± 0.02	n.d.	n.d.
	Total identified (%)			97.4	95.9	95.7	94.9	95.6	94.0
	Monoterpene hydrocarbons			9.6	6.8	5.5	-	17.6	18.7
	Oxygenated monoterpenes			84.8	85.3	86.5	91.1	76.3	75.3
	Sesquiterpene hydrocarbons			3.0	3.8	3.6	2.7	1.7	-
	Essential oil content (mL/100 g dry weight)			3.73	3.27	2.21	2.60	5.93	4.67

(a) Compounds are listed in the order of elution from an HP-5 MS capillary column; (b) AI: Arithmetic index determined relative to a homologous series of n-alkanes (C_9_–C_25_) on an HP-5 MS capillary column; (c) AI: Arithmetic index from literature data and Adams (2007) (d) n.d.: not detected.

## Data Availability

Not applicable.
